# Nanograss-Assembled NiCo_2_S_4_ as an Efficient Platinum-Free Counter Electrode for Dye-Sensitized Solar Cell

**DOI:** 10.3390/nano13212896

**Published:** 2023-11-02

**Authors:** Shada A. Alsharif

**Affiliations:** University College of Umlij, University of Tabuk, Tabuk 71491, Saudi Arabia; shalsharif@ut.edu.sa; Tel.: +966-54-334-3381

**Keywords:** dye-sensitized solar cell, electrodes, grass-like morphology, electrocatalysts, bimetallic sulfide

## Abstract

Dye-sensitized solar cells (DSSCs) are often viewed as the potential future of photovoltaic systems and have garnered significant attention in solar energy research. In this groundbreaking research, we introduced a novel solvothermal method to fabricate a unique “grass-like” pattern on fluorine-doped tin oxide glass (FTO), specifically designed for use as a counter electrode in dye-sensitized solar cell (DSSC) assemblies. Through rigorous structural and morphological evaluations, we ascertained the successful deposition of nickel cobalt sulfide (NCS) on the FTO surface, exhibiting the desired grass-like morphology. Electrocatalytic performance assessment of the developed NCS-1 showed results that intriguingly rivaled those of the acclaimed platinum catalyst, especially during the conversion of I_3_ to I^−^ as observed through cyclic voltammetry. Remarkably, when integrated into a solar cell assembly, both NCS-1 and NCS-2 electrodes exhibited encouraging power conversion efficiencies of 6.60% and 6.29%, respectively. These results become particularly noteworthy when compared to the 7.19% efficiency of a conventional Pt-based electrode under similar testing conditions. Central to the performance of the NCS-1 and NCS-2 electrodes is their unique thin and sharp grass-like morphology. This structure, vividly showcased through scanning electron microscopy, provides a vast surface area and an abundance of catalytic sites, pivotal for the catalytic reactions involving the electrolytes in DSSCs. In summation, given their innovative synthesis approach, affordability, and remarkable electrocatalytic attributes, the newly developed NCS counter electrodes stand out as potent contenders in future dye-sensitized solar cell applications.

## 1. Introduction

The rising consumption of fossil fuel-based energy has recently prompted considerable research into sustainable alternatives for future energy requirements [1,2,3,4,5]. Consequently, scientists have been delving into harnessing solar power by employing diverse photovoltaic devices which are capable of covering maximum solar spectrum and converting solar light to real electricity at low cost [4,5,6,7]. The dye-sensitized solar cells (DSSCs) have gained prominence owing to their cost-effectiveness, straight forward assembly method, and flexibility in tweaking their components. DSSCs represent a third-generation photovoltaic technology that offers advantages such as flexibility, potential cost-effectiveness, and simple fabrication processes compared to traditional silicon-based solar cells. The photoanode is essentially the heart of a DSSC [7,8,9,10]. It is responsible for absorbing sunlight and converting it into an electrical current. Here is how: The dye molecules are attached to the semiconductor material, typically titanium oxide or zinc oxide. When these dye molecules are hit by photons from sunlight, they become excited and inject electrons into the semiconductor material [2,10,11,12]. TiO_2_ is more commonly used due to its excellent stability, high refractive index, and strong bonding with dye molecules. However, ZnO has a faster electron mobility, which can potentially lead to better efficiency under certain conditions. The counter electrode, typically made of platinum, plays a critical role in the operation of the DSSC: Electron Collection. Once the electrons have travelled through the external circuit, generating electricity, they re-enter the cell at the counter electrode. It is the job of this electrode to collect these electrons and send them back to the electrolyte, completing the circuit. Platinum facilitates the reduction of the tri-iodide ions (I_3_^−^) back to iodide ions (I^−^) in the electrolyte [5,6,7,8,9]. This process is essential for the regeneration of the dye and the continuation of the electrical current. Platinum is chosen for this role primarily due to its exceptional conductivity, vast number of active sites for the redox reaction, and ease of deposition on conductive substrates. Its chemical stability also ensures a longer lifespan for the DSSC [12,13,14,15]. This is essentially the electrolyte solution in a DSSC. Here is its role: Once the dye has been excited by sunlight and has injected an electron into the TiO_2_ or ZnO, it is left in an oxidized state. To get back to its ground state and be ready to absorb more photons, it needs an electron. This electron is provided by the iodide ion (I^−^), which then becomes oxidized [1,2,3,4,10,11,12]. The oxidized iodide ions (usually in the form of I_3_^−^) then travel to the counter electrode, where they are reduced back to their original form, with the help of electrons that have come through the external circuit. The synchronized operation of the photoanode, platinum counter electrode, and redox mediator ensures efficient photon-to-electron conversion in a DSSC, offering a promising alternative to conventional silicon-based solar cells [15,16,17,18,19,20]. Nevertheless, despite platinum’s myriad benefits, challenges/difficulties like its susceptibility to corrosion during the redox process, high cost, and limited availability hinder its expensive use in DSSCs. This has sparked initiatives to unearth alternatives to Pt for counter electrode in DSSCs, including metal oxide, bimetallic oxides, sulfide, bimetallic sulfide, and metal nitrides, metal–polymer nanohybrids, etc. Notably, bimetallic sulfide, especially nickel- and cobalt-based bimetallic sulfide, has generated interest in DSSCs counter electrode application due to its easy preparation, low cost, uncomplicated synthesis, high conductivity, high surface area, large number of catalytic sites, and pronounced catalytic activity for the redox reaction during the electricity conversion process.

Pt has long been recognized as a benchmark catalyst due to its facile synthesis, ample catalytic sites, and robust conductivity [18,19,20,21,22,23]. However, despite its numerous merits over other metal catalysts, challenges such as its significant operational cost, rapid corrosion during redox reaction, and scarcity hinder its expensive deployment in solar cell application. In recent years, there has been a proliferation of research aimed at identifying suitable replacements for Pt, with a focus on both their cost-effectiveness and efficiency. Consequently, the quest for viable alternatives to expensive Pt-based counter electrodes has driven research towards diverse materials like metal and its binary structure of nitride, sulfide, oxide sulfide, metal oxides, and different types of nanohybrids for use in DSSCs [23,24,25]. Particularly, ternary metal sulfides, especially NiCoS_4_, have emerged as frontrunners for counter electrode applications. Their appeal stems from attributes like exceptional electrical conductivity surpassing both similar types of the metal sulfide and metal oxide merged in a ternary framework with commendable balance of the mechanical and thermal stabilities and superior properties including electrochemical and catalytic performances, a credit towards the presence of the two cations of the metal contribution in the cobalt and nickel framework [20,21,22,23,24,25,26,27]. The increased attention on NiCoS_4_ has seen several studies exploring different fabrication techniques and their efficiencies. In various works, DSSCs equipped with NiCoS4 counter electrodes have displayed efficiencies ranging from 4.5% to 7%, highlighting its potential as a viable alternative to Pt [28,29,30]. A notable method that has been previously employed is the hydrothermal method, which, while effective, has its own set of challenges and benefits [29,30]. The promise of NiCo_2_S_4_ has catalyzed the development of varied fabrication techniques for counter electrodes on FTO glass substrates, each bearing its unique pros and cons concerning cost, fabrication complexity, and environmental impact: for instance, the NiCoS_4_-based electrode prepared by the sulfide–argon gas blend to deposit the NiCoS_4_ for DSSCs application [15,16,17,18,19]. As well as the Pt–NiCoS_4_-based counter electrode prepared by the sputtering and pyrolysis method and used as a counter electrode in DSSCs. Given various backdrops, streamlining counter electrode fabrication by utilizing simple reactants and a solitary process is vital for large scale production [26,27,28,29,30,31]. Hence, an approach that simplifies synthesis, eschewing complex chemicals and multistep procedures could offer notable benefits for directly growing NiCoS_4_ on FTO glass substrate, minimizing potential film detachment issues. This research, therefore, zeroes in on the direct cultivation of NiCoS_4_ on fluorine-doped coated glass using a simplified, singular solvothermal process, augmented by the addition of surfactant guiding agent. Post fabrication, the prepared electrode underwent rigorous characterization through various spectroscopic and microscopic methodologies, followed by integration and testing within a DSSC setup. Preliminary findings indicate that the fabricated counter electrode boasts a power conversion efficiency of 6.60%, a figure that commendably parallels the 7.19% efficiency of its Pt counterpart in a similar DSSC configuration.

## 2. Materials and Methods

### 2.1. Materials

Hexa-methylene tetramine, 1-propyl-3-methylimidazolium iodide, iodine, 4-tert-butylpyridine, lithium iodide, cis-bis (isothiocyanato)-bis(2,20-bipyridyl-4,40-dicarboxylato)-ruthenium (II) bis-trabutylammonium, and acetonitrile were sourced from Sigma-Aldrich (St. Louis, MO, USA). The cobalt nitrate, thiourea, detergent, and nickel nitrate were acquired from Sigma-Aldrich. The TiO_2_ paste was procured from Dyesol Industries Pty. Ltd., Sydney, Australia. The fluorine-doped tin oxide (FTO) was sourced from NSG Japan (Tokyo, Japan).

### 2.2. Methods

To investigate the structure and surface design of the NCS-1 and NCS-2 layered over the FTO, field emission scanning electron microscopy (JEOL-JSM-6700, JEOL, Akishima, Japan) and X-ray diffraction (Bruker, Mannheim, Germany) were performed. The chemical stated and its composition examined through the Thermo Scientific X-ray photoelectron spectroscopy (Thermo Scientific, Waltham, MA, USA). Artificial solar illumination was generated using a calibrated solar simulator (Oriel Sol 3A, Newport, Irvine, CA, USA) equipped with a Xenon lamp. The source meter (Keithley-2400, Keithley, Cleveland, OH, USA) integrated with specialized software was utilized to check the current density versus voltage under dark and illumination condition using Newport sola simulator of the developed DSSC cell. A mixture of the 0.6 M 1-propyl-3-methylimidazolium iodide, 0.1 M lithium iodide, 0.03 M iodine, and 0.05 M 4-tert-butylpyridine was prepared in acetonitrile as a solvent. The counter electrodes we prepared, specifically NCS-1 and NCS-2, were subjected to rigorous cyclic voltammetry testing. For these tests, the scan rate was set at 50 mV/s. Within the experimental setup, NCS-1 and NCS-2 were utilized as the working electrodes, while a platinum (Pt) wire served as the counter electrode. It is essential to note that the choice of Pt wire as the counter electrode offers a reliable and consistent standard for comparison, given its widespread acceptance in electrochemical analysis. All electrochemical measurements, including potential sweeps and current recordings, were conducted using a Metrohm Autolab electrochemical workstation (Utrecht, The Netherlands). This workstation, equipped with a potentiostat, ensured precision and accuracy in capturing the electrochemical behavior of the prepared counter electrodes.

### 2.3. Material Synthesis

#### 2.3.1. Counter Electrode Fabrication on FTO-Coated Glass

The FTO underwent an ultrasonic cleaning process involving a sequence of detergent, ethanol, and water. Following this, precise quantities of the nickel nitrate (0.1 M) and cobalt nitrate (0.2 M) were dissolved in DI water and ethanol. The above reaction solution was further stirred for 20 min until all the precursor mixed well. The directing agent of hexa-methylene tetramine and thiourea (SC(NH_2_)_2_) was later added to the solution. This mixture was then placed into a Teflon-lined autoclave, with the FTO glass positioned strategically inside (in such way that the conductive surface faced downward). Following which was heat treatment at 170 °C in an electric oven for 12 h. The reaction vessel was cooled down to room temperature naturally and after that the sample was collected, rinsed with water and ethanol to remove the unreacted precursor part, and dried at 60 °C in oven overnight. The NiCo_2_S_4_ synthesized over FTO abbreviated as NSC-1 and NCS-2 depends on the concentration of the hexa-methylene tetramine (0.05 and 0.1 M) directing agent. The suggested formation mechanism could be written as follows.

Ni(NO_3_)_2_ + Co(NO_3_)_2_ + EtOH/H_2_O → Ni^2+^ + Co^2+^ + OH^−^

2Co(NO_3_)_2_ + Ni(NO_3_)_2_ + 4S → NiCo_2_S_4_ + 6NO_2_ + 2O_2_

SC(NH_2_)_2_ → NH_2_CN + H_2_S

Ni^2+^ + Co^2+^ + OH^−^ → Ni-Co_2_OOH + H_2_O

Ni-Co_2_OOH + H_2_S → NiCo_2_S_4_ + H_2_O

#### 2.3.2. Preparation of Photoanode and DSSC Assembly

Commercial FTO-coated glass was meticulously cleaned using an ultrasonication process. Subsequently, this glass was layered with TiO_2_ paste via sharp blades. To form a TiO_2_ compact layer, the glass was dipped into a TiF_4_ solution and treated at 70 °C for 30 min. This is followed by heating the glass at 450 °C for an hour to enhance the crystallinity of the TiO_2_ layer. Before applying the TiO_2_ paste, unwanted areas on the glass are shielded using 3 M magic tape. The coated FTO glass with TiO_2_ was then dipped in a dye solution for 12 h at 40 °C. The dye-soaked electrode was then paired with a counter electrode, sealed, and finally filled with the formulated electrolytes. The film undergoes a post-treatment in a TiF_4_ solution, identical to the initial pretreatment step. The treated electrodes are then immersed in an N719 dye solution in absolute ethanol. This is left at room temperature overnight in a dark environment. Visualize a DSSC as a composite system comprising two electrically conductive electrodes, sandwiching an electrolyte with a redox duo, specifically iodide/triiodide ions. These electrodes rest on glass layers coated with a transparent conductive oxide. The operational electrode features a TiO_2_ layer that is sensitized using specific dyes.

## 3. Results and Discussion

### 3.1. Formation Mechanism

**Figure 1:**
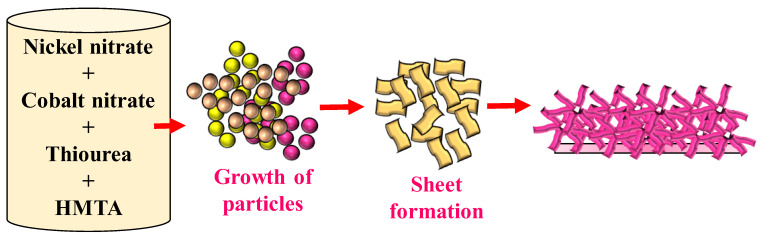
Suggested schematic of the growth of the NCS-1 over the FTO substrate.

The specific pathway of formation for these materials remains somewhat elusive, yet several factors, such as the nature of the precursor, the temperature, and the formation of complexes, play undeniable roles in determining the size, shape, and phase of the resulting materials. At the onset, under solvothermal conditions, cobalt and nickel ions engage in complex formation with thiourea, leading to the generation of rudimentary NiCo_2_S_4_ particles, as illustrated in Figure 1. These primary particles coalesce, adopting a grass-like nanostructure. It is theorized that as the process progresses, nanoparticles tend to gravitate around gas bubbles, giving rise to the initial stages of nanocluster formation. These emerging clusters, influenced by the inherent magnetic interactions among particles, begin to elongate, forming string-like aggregates. These elongated aggregates subsequently serve as platforms or ‘nucleation sites’ where further accretion occurs, eventually leading to the development of nanosheets. Due to their intrinsically elevated surface energy, these nanosheets tend to attract each other, culminating in intricate, reminiscent structures. On a related note, upon increasing the temperature, thiourea is subject to hydrolysis. This chemical breakdown results in the liberation of CO_2_ and NH_3_ gases. Intriguingly, the evolving gas bubbles appear to act as templates or molds, playing a pivotal role in dictating the final morphology of the synthesized materials. This templating effect of gas bubbles offers a potential avenue to control the shape and structure of materials in future synthetic endeavors.

### 3.2. Structural and Morphological Studies

**Figure 2:**
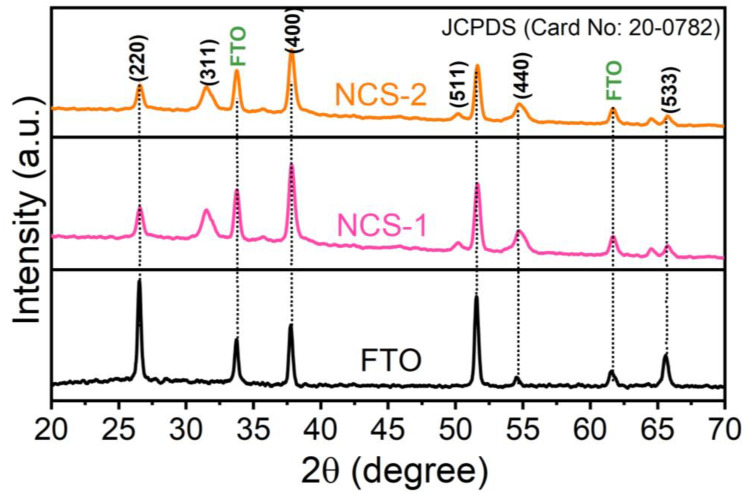
XRD pattern of the NCS-1, NCS-2, and FTO substrate.

The crystallinity and structural integrity of NCS-1 and NCS-2 when deposited on the FTO were rigorously evaluated using X-ray diffraction (XRD) techniques. The results are visualized in Figure 2. Delving into this figure allows for a juxtaposition between the XRD patterns of the pristine FTO and those of FTO when embellished with NCS-1 and NCS-2. Distinctive diffraction peaks emerge at 2θ values of 26.81, 31.57, 38.0, 32.0, and 55.30°. These peaks are not arbitrary; they correspond with crystallographic planes denoted as (111), (220), (311), (400), (422), (511), (440), (533), and (444). A deeper dive into the literature references, particularly those indexed by JCPDS (Card No: 20-0782), aligns these peaks and planes with the inherent cubic structure characteristic of both NCS-1 and NCS-2. In essence, these congruencies between observed diffraction peaks and the known crystalline planes for this compound type serve as solid indicators. They reinforce the notion that the NCS-1 and NCS-2 were indeed successfully anchored onto the FTO substrate. Furthermore, the consistency of the patterns suggests a homogenous and well-ordered crystalline deposition, which is crucial for effective material performance.

**Figure 3:**
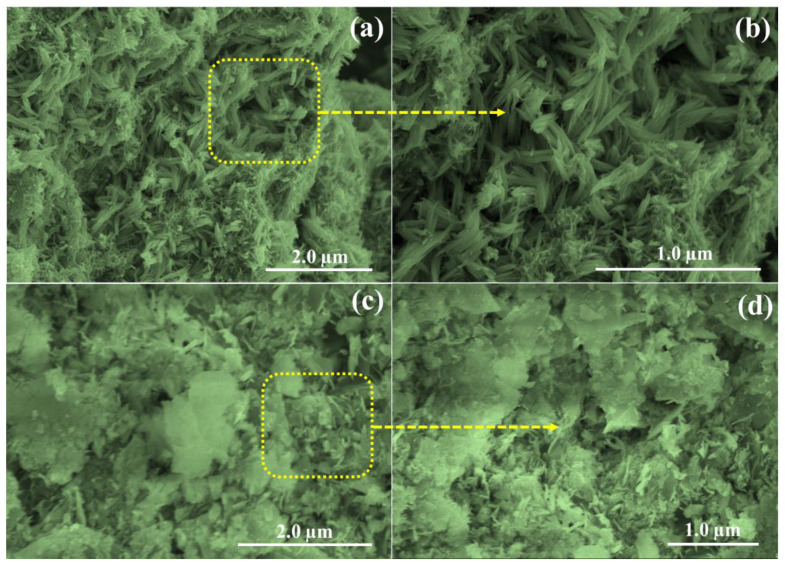
(**a**,**b**) SEM images of the NCS-1 at different magnification. (**c**,**d**) SEM images of the NCS-2 at different magnification.

The intricate details of the surface morphology of the synthesized NCS-1 electrode were examined using field-emission scanning electron microscopy (FESEM), and the compelling visuals can be seen in Figure 3a–d. The morphology, which intricately influences the catalytic action and surface properties of materials, carries profound significance for the overall performance of the electrode. Upon examining Figure 3, one is immediately drawn to the unique, slender grass-like structures of NCS-1. These structures are not isolated; they appear to intertwine and link at various junctions across the surface. A closer, more discerning look emphasizes that these grass-like protrusions are not only interconnected but also maintain a level of structural cohesion. This interlinked framework augments the electrode’s conductive behavior. Essentially, the intertwined nature ensures a continuous path for electron transport, reducing resistances and enhancing conductivity, a factor that is pivotal for efficient electrode performance. Furthermore, the dense and connected nature might aid in providing a larger surface area, promoting greater interaction with the surrounding medium, which can be crucial in various electrochemical applications. The scanning electron microscopy images of NCS-2 show that the structure resembling grass is not distinct (Figure 3c,d). This means that instead of individual “grass-like” structures, the segments appear to merge together. This amalgamation can be attributed to the insufficient quantity of the directing agent used during the preparation. When the directing agent is not present in adequate amounts, it cannot guide the assembly of the structures effectively. As a result, the electrode performance diminishes because clear pathways, which are crucial for efficient conductivity and electron transfer, are obstructed. In simpler terms, think of it like building a pathway in a garden. If you do not have enough guidance or material to create distinct paths, the plants might grow everywhere and block the way, making it difficult to walk through. Similarly, the electrode’s performance is compromised due to the lack of clear structural pathways.

**Figure 4:**
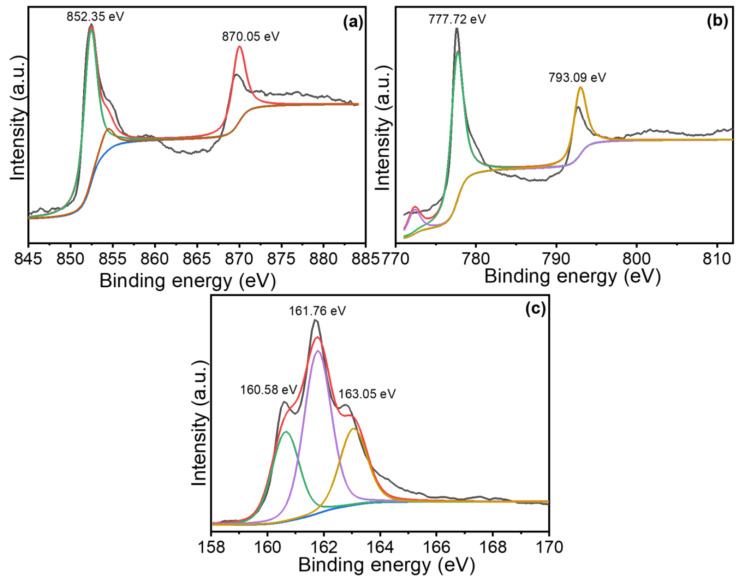
XPS high resolution spectra (**a**) Ni 2p, (**b**) Co 2p, and (**c**) S 2p of the NCS-1 electrode.

The XPS was utilized to delve into the chemical constituents of the NCS-1 over the FTO, with the results visualized in Figure 4. Transitioning to Figure 4a, the Ni 2p spectra brings forth two distinct peaks at BE value of 852.35 eV and 870.05 eV. These can be ascribed to the Ni 2p_1/2_ and Ni 2p_3/2_ electronic state, respectively [18,19,29,31]. This 18 eV difference in the BE is consistent with the spin-orbit splitting of the Ni 2p electron level. A closer look at Figure 4b shows the peak at 777.72 eV, which can be attributed to the Co 2p_3/2_ core level of cobalt. Typically, a binding energy around this range suggests the presence of cobalt in an oxidized state, potentially Co(II) or Co(III), depending on the chemical environment. The peak at 793.09 eV corresponds to the Co 2p_1/2_ core level. The approximately 15 eV difference between the peak is consistent with the spin-orbit splitting observed for cobalt in many of its compounds [20,22,25]. Moreover, a comprehensive examination of the S 2p spectra, illustrated in Figure 4c, reveals both a principal peak and associated satellite peak at BE values of 161.76 eV, 163.05 eV, and 160.58 eV. The observed BEs in the S 2p region for the NiCo_2_S_4_ sample suggest a complex sulfur environment, with predominant sulfide character along with potential variations in the local coordination environment or surface-oxidized species. The occurrence of these peaks is indicative of both metal-sulfur and cobalt sulfur bonds within the structure. Notably, these findings align well with data presented in prior research [20,21,22,23,24,25,26,27], adding further credibility to our observation and discussion.

**Figure 5:**
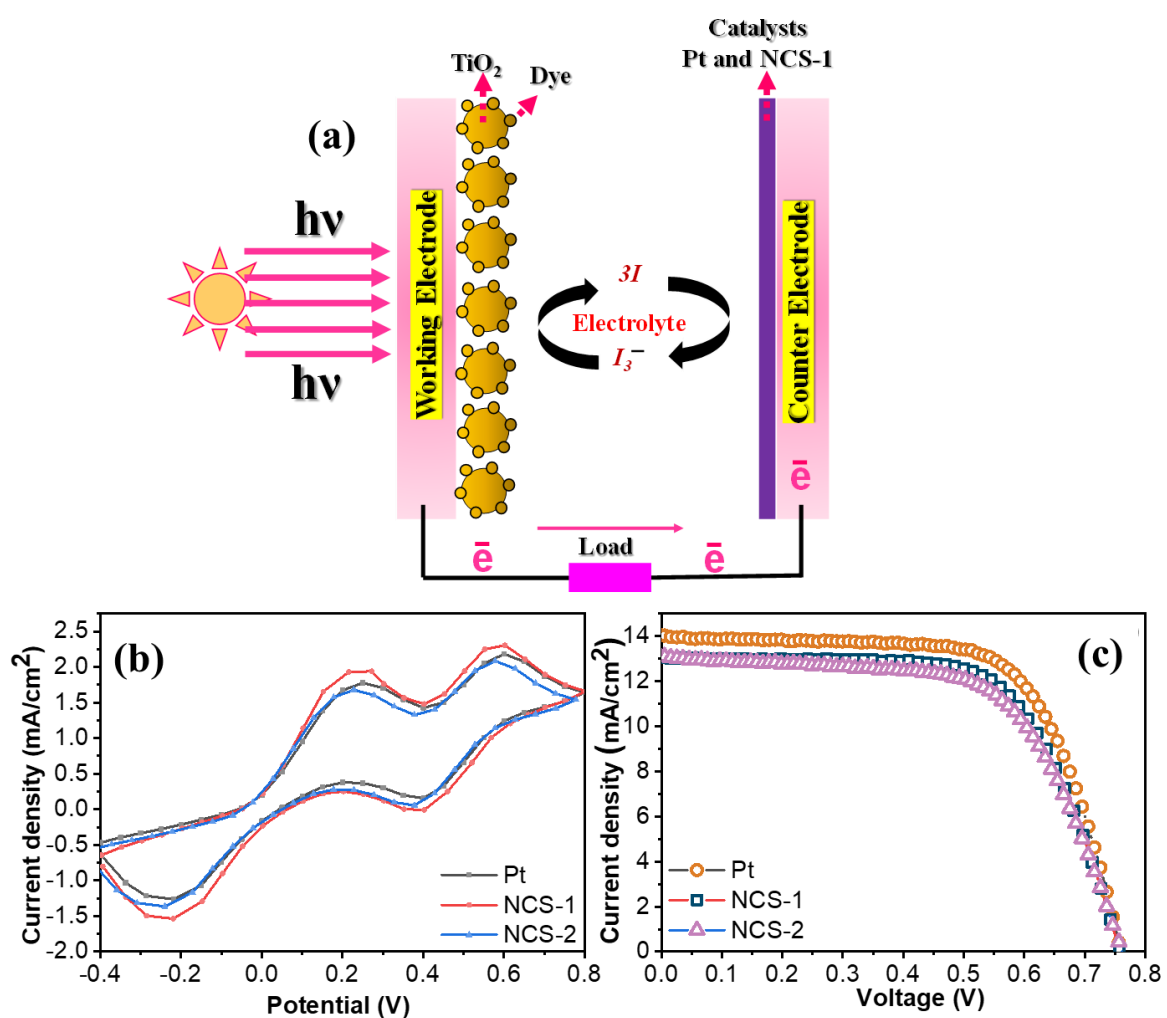
(**a**) Schematic image of the assembled DSSC, (**b**) cyclic voltammograms of the Pt, NCS-1, and NCS-2, and (**c**) current density–voltage graph of the Pt, NCS-1, and NCS-2.

The efficiency and attributes of the DSSC are significantly determined by the choice of materials used in its various components, as illustrated in Figure 5a. Delving into the cyclic voltammetry results for both the Pt and NCS electrodes offers a comprehensive understanding of their electrocatalytic behaviors. In particular, these results shed light on the redox properties of the catalysts in relation to the sensitizing dyes, as seen in Figure 5b [25,26,27,28,29,30,31]. The positive potential in the cyclic voltammetry curve is indicative of the redox reaction involving I_2_/2I_3_^−^. Conversely, the negative potential mirrors the redox dynamics of I^−^/I_3_^−^. These observed phenomena can be better understood through the ensuing equation. Furthermore, the relative positions and magnitudes of these potentials provide insights into the efficiency of electron transfer and the potential for recombination. An electrode material with rapid redox reactions ensures swift electron transfer, reducing energy losses and thereby improving overall device performance [23,25,29].

2I_3_^−^ = 3I_2_ + 2e^−^

3I^−^ = I_3_^−^ + 2e^−^

The ability of the NCS-1 at the surface of the FTO substrate to reduce the I_3_^−^ to I^−^ was analyzed using cyclic voltammetry. The graph of the Pt and NCS electrodes revealed two evident peaks representing the oxidation and reduction process that occurred during the electrocatalytic reactions. Notably, the negative pair represents the redox conversion of I^−^/I_3_^−^ while the positive pair indicates the I_2_/I_3_^−^ transition during electrocatalytic reaction (Figure 5b). The graph also displays that the peak current in the case of the NCS is almost the same compared to the Pt electrode which suggests that the fast reduction rate is similar for the NCS-1 electrode [12,13,14,15,16,17]. The peak-to-peak separation and related current density (*E_pp_*) can be correlated with the faster electron transfer kinetics. The NCS-1 electrode has a slightly lower Epp than the Pt electrode which suggests that enhanced kinetics led to better electrocatalytic activity towards the lithium iodide electrolytes [28,29,30,31]. The above results showed that the NCS-1 counter electrode displayed similar catalytic activity to the Pt counter electrode for the reduction of the I_3_^−^ to I^−^. A pivotal feature is the unique grass-like pattern over the FTO substrate which provides multiple benefits such as high surface area, short pathway towards the charge transfer kinetic process, and numerous catalytic sites which lead to swift the electron transfer [30,31]. The stability of NiCo_2_S_4_ electrodes is crucial for their electrochemical applications [32,33,34,35,36,37,38]. NiCo_2_S_4_’s unique structure offers resistance to mechanical and electrochemical stresses, ensuring longevity. Its reduced volume changes during cycling enhance stability compared to binary sulfides. Additionally, when paired with the right electrolyte, it can form a stable solid–electrolyte interphase, further improving its performance. The literature also indicates that nanostructured NiCo_2_S_4_ exhibits superior durability and efficiency. Overall, our prepared NiCo_2_S_4_ electrodes show promising stability, aligning with the existing literature. The stability of the NCS-1 (Figure S1) counter electrode was thoroughly evaluated. The results revealed that the electrode remained responsive after 20 cycles, indicating its durability and consistent performance. This means that even after being used multiple times (20 cycles in this context), the electrode did not degrade or lose its effectiveness. Furthermore, this behavior of maintaining its characteristics was observed even after prolonged usage, suggesting that the electrode is not only stable but also has a longer operational lifespan. In simpler terms, just like a rechargeable battery that holds its charge even after being used many times, the NCS-1 counter electrode continues to perform well over repeated cycles, showcasing its reliability and potential for extended applications.

Switching focus to the photovoltaic attributes of all the counter electrodes (Pt@FTO, NCS-1@FTO, and NCS-2@FTO) within the dye-sensitized solar cell, a DSSC device assembly fabricated using the abovementioned counter electrode and corresponding photo current density voltage curve are represented in Figure 5c. The comparative analysis of the photo current density voltage curve infers that the NCS-1 and NCS-2 counter electrodes’ performance is comparable to the well-known Pt-based CE of the DSSC. The important parameters of the photovoltaic such as open circuit voltage (*V_oc_*), short circuit current density (*J_sc_*), fill factor (*FF*), and photoelectric conversion efficiency (*PCE*) are listed in Table 1.

In the assembly of DSSCs using NCS-1 and NCS-2, the observed values for *V_oc_* were 0.75 V and 0.74 V, respectively, with fill factors (*FF*) being 66.56 and 63.21, and current densities (*J_sc_*) at 13.05 and 13.05 mA·cm^−2^. When compared to the performance of the platinum (Pt) counter electrode, which exhibited a *V_oc_* of 0.75 V, an *FF* of 67.68, and a *J_sc_* of 13.98 mA·cm^−2^, the metrics are strikingly similar. The comparable values between NCS-1 and NCS-2 can be attributed to their analogous electrocatalytic behaviors. Specifically, both counter electrodes exhibited consistent catalytic activity in facilitating the reduction of I_3_^−^ ions, leading to increased production of I^−^ ions. This, in turn, promotes efficient regeneration of the dye, thereby enhancing the device’s ability to capture light and effectively inject electrons, optimizing the performance of the DSSC. Furthermore, these findings are corroborated by the results from cyclic voltammetry (CV) tests, reinforcing the notion of comparable catalytic activities between the two materials. The DSSCs utilizing NCS-1 and NCS-2 as counter electrodes achieved photoelectric conversion efficiencies of 6.60% and 6.29%, respectively. These values closely approach the efficiency of 7.19% achieved with the benchmark Pt counter electrode. Such performance metrics underscore the potential of NCS materials as viable alternatives to traditional Pt counter electrodes in DSSC applications, paving the way for more cost-effective and sustainable photovoltaic solutions. The table presents a comprehensive comparison of the performance metrics of various dye-sensitized solar cell electrodes (Table S1). The most frequently assessed material, NiCo_2_S_4_, displays variable performance across different studies as tabulated in Table S1. The introduction of reduced graphene oxide into the NiCo_2_S_4_ matrix seems to offer an enhanced power conversion efficiency, signifying the synergistic effect of combining materials to optimize electron transport properties. Other materials like CoNi_2_S_4_ and CuInS_2_ also exhibit competitive performance, underscoring the rich landscape of potential electrode materials for DSSCs. Of particular note is the achievement in the present work with NiCo_2_S_4_, which approaches the highest efficiency levels, suggesting advancements in electrode fabrication or treatment that maximize the potential of this material.

## 4. Conclusions

In this study, we developed a simplified solvothermal technique to grow a distinct grass-like pattern on fluorine-doped tin oxide, which was designed to act as a counter electrode in dye-sensitized solar cell assembly. The examination of the morphology and structure confirmed the successful deposition of NCS on the FTO substrate surface with grass-like morphology. The electrocatalytic results displayed that the NCS-1 showed similar catalytic behavior towards the I^3−^ and I^−^ conversion as compared to the Pt counter electrode. The assembled DSSC assembly with NCS-1 and NCS-2 counter electrode produced power conversion efficiency of 6.60% and 6.29% which are close to the Pt counter electrode efficiency (7.19%). The comparative efficiency of the NCS-1, NCS-2, and Pt counter electrode could be due to the thin, sharp grass-like structure. This provides a large number of catalytic sites and high surface area, as identified through scanning electron microscopy, which are essential for the electrocatalytic reactions. Given its easy fabrication, cost effectiveness, and electrocatalytic activity performance, NCS emerges as a promising counter electrode material for dye-sensitized solar cell application. 

## Figures and Tables

**Table 1:** Photovoltaic performance summary of the Pt, NCS-1, NCS-2 assembled DSSC devices.

Electrodes	*V_oc_* (V)	*J_sc_* (mA·cm^−2^)	*FF*	*PCE* (%)
**Pt**	0.75	13.98	67.68	7.19
**NCS-1**	0.75	13.05	66.56	6.60
**NCS-2**	0.74	13.04	63.21	6.29

## Data Availability

The data that support the findings of this study are available from the corresponding author upon reasonable request.

## Supplementary Materials

The following supporting information can be downloaded at: https://www.mdpi.com/article/10.3390/nano13212896/s1, Figure S1: The cyclic voltammetry (CV) was used to evaluate the NCS-1 counter electrode over 20 cycles; Table S1: Comparative Analysis of DSSC Electrode Materials: NCS vs. Alternatives. Refs. [15,16,28,35,36,37,38] are cited in the supplementary materials.
