# Supplementary material for: Nanograss-Assembled NiCo2S4 as an Efficient Platinum-Free Counter Electrode for Dye-Sensitized Solar Cell
Source: Nanomaterials (Basel). 2023 Nov 2;13(21):2896. doi: 10.3390/nano13212896 (PMC10650019; doi:10.3390/nano13212896)
Supplement: Supplementary file 1 [file nanomaterials-13-02896-s001.zip › nanomaterials-2670147-supplementary.pdf]

ESI

Nanograss-Assembled $NiCo_2S_4$ as An Efficient Platinum-Free Counter Electrode for Dye-Sensitized Solar Cell

Shada A. Alsharif

University College of Umlij, University of Tabuk, Tabuk 71491, Saudi Arabia; shalsharif@ut.edu.sa;

Tel.: +966-54-334-3381

The cyclic voltammetry (CV) was used to evaluate the NCS-1 counter electrode over 20 cycles

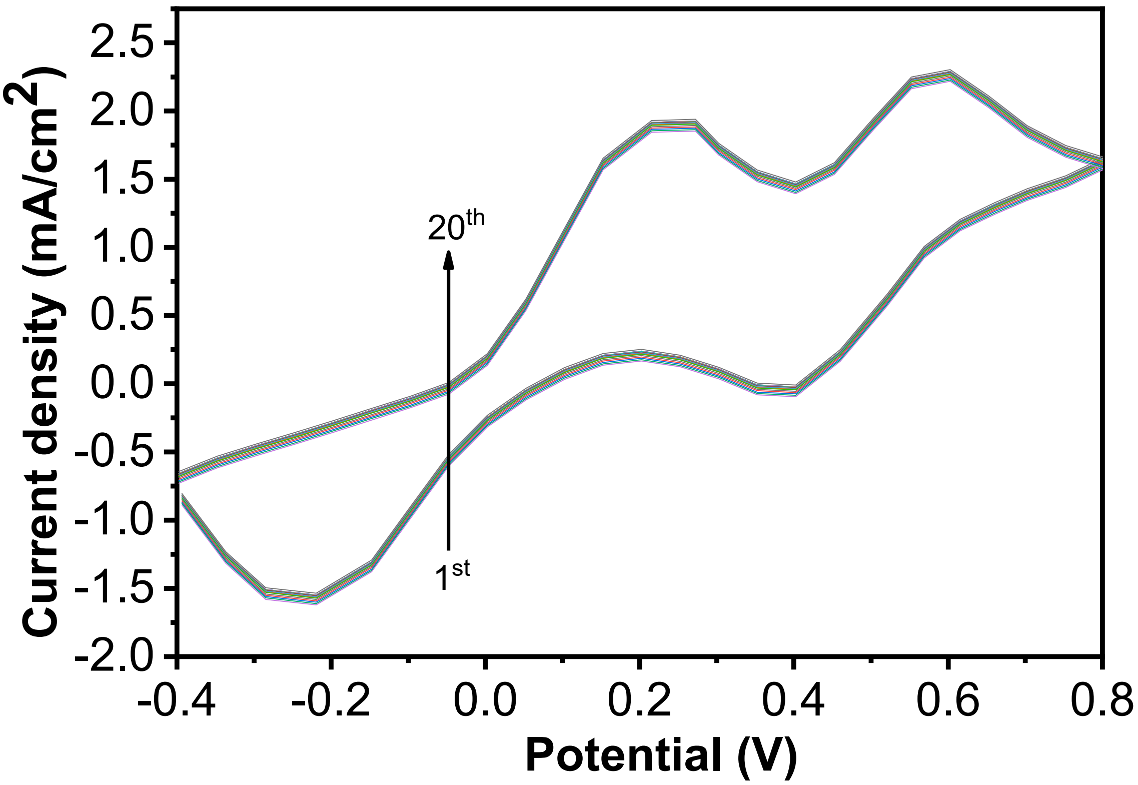

Figure S1. The cyclic voltammetry (CV) was used to evaluate the NCS-1 counter electrode over 20 cycles.

Table S1: Comparative Analysis of DSSC Electrode Materials: NCS vs. Alternatives

Electrode materials	V_{oc} (V)	PCE (%)	References
$NiCo_2S_4$	0.830	6.51	S1
$NiCo_2S_4$	0.755	4.73	S2
$NiCo_2S_4$/RGO	0.742	6.0	S2
$NiCo_2S_4$	0.76	5.81	S3
$CoNi_2S_4$	0.75	4.61	S4
$CuInS_2$	0.78	5.79	S5
$NiCo_2S_4$	0.55	4.22	S6
$NiCo_2S_4$	0.69	5.24	S7
$NiCo_2S_4$	0.75	6.60	Present work

References

S1. Ling Li, Xue Zhang, Shuang'an Liu, Baolai Liang, Yucang Zhang, Wenming Zhang, One-step hydrothermal synthesis of NiCo2S4 loaded on electrospun carbon nanofibers as an efficient counter electrode for dye-sensitized solar cells, Solar Energy, Volume 202, 2020, Pages 358-364,

S2. Hui Nan, Jianhua Han, Qiang Luo, Xuewen Yin, Yu Zhou, Zhibo Yao, Xiaochong Zhao, Xin Li, Hong Lin, Economically synthesized NiCo2S4/reduced graphene oxide composite as efficient counter electrode in dye-sensitized solar cell, Applied Surface Science, Volume 437, 2018, Pages 227-232.

S3. Kangle Liu, Aixiang Wei, Jun Liu, Zhen Liu, Zhiming Xiao, Yu Zhao, NiCo2S4 nanosheet thin film counter electrodes prepared by a two-step approach for dye-sensitized solar cells, Materials Letters, Volume 217, 2018, Pages 185-188.

S4. Shi, Z.; Deng, K.; Li, L. Pt-free and efficient counter electrode with nanostructured CoNi2S4 for dye-sensitized solar cells. Sci. Rep. 2015, 2-7.

S5. Wang, L.; He, J.; Zhou, M.; Zhao, S.; Wang, Q.; Ding, B. Copper indium disulphide nanocrystals supported on carbonized chicken eggshell membranes as efficient counter electrodes for dye-sensitized solar cells. J. Power Sources 2016, 315, 79–85.

S6. Junwu Xiao, Xianwei Zeng, Wei Chen, Fei Xiao, and Shuai Wang, High electrocatalytic activity of self-standing hollow NiCo2S4 single crystalline nanorod arrays towards sulfide redox shuttles in quantum dot-sensitized solar cells, Chem. Commun., 2013,49, 11734-11736. DOI: 10.1039/c3cc44242j.

S7. Chenle Zhang, Libo Deng, Peixin Zhang, Xiangzhong Ren, Yongliang Li & Tingshu He, Electrospun NiCo2S4 with extraordinary electrocatalytic activity as counter electrodes for dye-sensitized solar cells. J Solid State Electrochem 21, 3579–3588 (2017).
